# Reimagining Identity in Postcolonial East African Literature: A Comparative Analysis of Ngugi wa Thiong'o and Abdulrazak Gurnah

**DOI:** 10.12688/f1000research.167352.4

**Published:** 2026-03-19

**Authors:** Bushra Juhi Jani

**Affiliations:** 1 Supporting Sciences Unit/ College of Medicine, Al-Nahrain University, Baghdad, Baghdad Governorate, 1100, Iraq

**Keywords:** Ngugi wa Thiong'o, Abdulrazak Gurnah, postcolonial literature, East Africa, historical trauma, intergenerational memory, exile, resistance, hybridity, decolonization, language politics, narrative ethics

## Abstract

This essay compares how Ngugi wa Thiong’o and Abdulrazak Gurnah, two leading East African writers, represent postcolonial identity and historical trauma through fiction. The comparison is justified by both authors’ shared East African context and their divergent responses to colonialism: Ngugi embraces radical language politics, writing in Gikuyu as an act of decolonisation and foregrounding collective cultural reclamation, while Gurnah employs narrative ambiguity, exile experience, and linguistic hybridity to explore individual psychological complexity. These contrasting strategies constitute two distinct but complementary models of postcolonial literary ethics. The analysis proceeds through three interconnected themes: the representation of historical trauma in narrative structure; the function of collective memory as both burden and resistance; and the transmission of intergenerational trauma through storytelling and language. It then examines their divergent approaches to language politics and narrative authority, their representations of exile and return, and their distinct visions of postcolonial literary ethics. Despite their differences, Ngugi prioritizing political clarity and revolutionary consciousness, Gurnah exploring subtle psychological details and narrative dissonance, both authors demonstrate literature’s ethical power to contest historical erasure and reimagine postcolonial futures with compassion and complexity. Together, they reveal the diversity within postcolonial African literature rather than assuming a monolithic ‘African’ response to colonialism.

## Introduction

In the aftermath of East African independence movements, literature emerged not merely as artistic expression but as a vital site for reimagining identity, confronting historical trauma, and negotiating the complex legacies of colonialism. This essay examines how two of the region’s most significant writers—Ngugi wa Thiong’o of Kenya and Abdulrazak Gurnah of Tanzania—have approached these challenges through radically different yet equally profound literary strategies. Both authors write from the fractured terrain of postcolonial experience, but their divergent approaches to language, memory, and resistance illuminate the varied paths through which literature confronts colonial wounds and envisions decolonial futures.

The justification for placing these authors in comparative dialogue is both contextual and theoretical. Both are East African writers whose careers span the post-independence era; both engage with colonialism’s long afterlives through fiction; yet they diverge sharply in their aesthetic and political commitments — Ngugi foregrounding collective mobilisation and linguistic decolonisation, Gurnah exploring individual interiority, exile, and moral ambiguity. This divergence itself constitutes the comparative essay’s primary analytical object, revealing the diversity within postcolonial African literary practice rather than assuming a monolithic response to colonial legacies. Key similarities include: both authors’ engagement with intergenerational trauma, memory as a contested site, and the ethics of narrative witnessing. Key differences include: Ngugi’s collectivist, politically explicit aesthetics versus Gurnah’s individualist, psychologically ambiguous narrative style; Ngugi’s commitment to indigenous language versus Gurnah’s transformation of English from within.

This comparative analysis sits at the intersection of postcolonial literary studies, trauma theory, and memory studies, drawing additionally from anthropological and historical perspectives on East African societies. By examining these authors together—one who has been canonized in postcolonial studies for decades (Ngugi) and another whose recognition through the 2021 Nobel Prize affirms his significant yet distinct contribution (Gurnah)—we gain deeper insight into the multifaceted nature of postcolonial literary resistance ( Gurnah 2021b).

The central question guiding this analysis is: How do Ngugi and Gurnah’s contrasting narrative approaches to historical trauma, memory, and identity construction reflect different possibilities for postcolonial literary ethics and politics? This comparison matters because it reveals the diversity within postcolonial African literature rather than assuming a monolithic “African” response to colonialism. As Simon Gikandi argues that the task of reading “other” literatures is to recognize their plurality and complexity while also acknowledging their unique historical and cultural contexts ( 2001, 4). While much scholarship has examined each author individually, few studies have placed them in direct conversation to illuminate their complementary insights. By placing Ngugi and Gurnah in sustained comparative dialogue, this study demonstrates how their differing narrative strategies produce distinct yet equally significant ethical responses to historical violence.

The researcher argues that Ngugi and Gurnah represent two ethical poles in postcolonial literary practice: Ngugi embraces an aesthetics of clarity and collective mobilization that foregrounds political agency, while Gurnah cultivates an aesthetics of ambiguity and individual interiority that emphasizes psychological complexity. These approaches reflect not simply personal preferences but profound philosophical divergences about how literature should respond to historical violence and what forms decolonization might take in both politics and aesthetics.

This study contributes to existing scholarship by demonstrating how Ngugi and Gurnah articulate contrasting yet complementary models of postcolonial ethics—one grounded in linguistic decolonization and collective political agency, the other in narrative ambiguity and ethical witnessing. By placing these authors in sustained comparative dialogue across the themes of trauma, memory, language, and exile, the paper moves beyond author-specific readings to illuminate broader questions about how postcolonial literature negotiates responsibility, identity, and historical violence.

As Achille Mbembe explains in his examination of postcolonial subjectivity, “all human societies participate in a complex order, rich in unexpected turns, meanders, and changes of course” ( 2001, 14). This complexity defies linearity, embracing a multiplicity of paths and temporalities shaped by both internal forces and external domination. Both Ngugi and Gurnah navigate this entanglement, but they chart different paths through it-Ngugi seeking clarity and coherence, Gurnah embracing contradiction and ambivalence.

The analysis unfolds through three interconnected themes: first, how historical trauma shapes narrative structure and voice; second, how collective memory functions as both burden and resistance; and third, how intergenerational trauma is transmitted and potentially transformed through storytelling. I will then explore the authors’ divergent approaches to language politics and narrative authority, their representations of exile and return, and finally, their distinct visions of postcolonial literary ethics.

Each section draws from close readings of primary texts, including Ngugi’s *A Grain of Wheat* ( 1967), *Petals of Blood* ( 1977), and *Matigari* ( 1987) alongside Gurnah’s *Paradise* ( 1994), *By the Sea* ( 2021a), and *Afterlives* ( 2020), among others. These readings are informed by theoretical frameworks from postcolonial studies, trauma theory, and narrative ethics, including the work of Frantz Fanon, Homi Bhabha, Gayatri Spivak, and Cathy Caruth, while remaining attentive to the specific cultural and historical contexts of East Africa.

This study adopts a qualitative comparative literary analysis grounded in close textual reading. The selected novels were chosen because they represent key phases in each author’s engagement with colonial and postcolonial history: Ngugi’s works foreground moments of anticolonial struggle and post-independence disillusionment, while Gurnah’s novels explore exile, memory, and ethical ambiguity across colonial and postcolonial transitions. Reading these texts comparatively allows for an examination of how differing narrative strategies, language choices, and ethical positions shape postcolonial identity formation in East African literature.

Through this comparative approach, the researcher hopes to contribute to a more sophisticated understanding of how postcolonial literature engages with historical trauma and identity formation in ways that transcend simple binaries of resistance and complicity, tradition and modernity, or local and global. As Gikandi reminds us, African literature has always been shaped by the complicated relationship between the aesthetics of the text and the politics of context ( 2001, 9). By examining how Ngugi and Gurnah negotiate this relationship differently, we gain insight into the diverse ethical possibilities that postcolonial literature offers for addressing the wounds of history and imagining alternative futures.

Ngugi wa Thiong’o’s novels examined in this study— *The River Between*, *A Grain of Wheat*, *Petals of Blood*, and *Matigari*—span the period from early colonial intrusion to post-independence disillusionment in Kenya. Collectively, these works trace the psychological, cultural, and political consequences of colonial domination, focusing on communal resistance, betrayal, and the unfinished promises of liberation.

Abdulrazak Gurnah’s novels— *Paradise*, *By the Sea*, *Desertion*, and *Afterlives*—explore the long afterlives of colonialism in East Africa, particularly through experiences of displacement, exile, and fragmented memory. Set across German and British colonial periods and their aftermaths, these texts foreground individual lives shaped by historical violence, migration, and ethical uncertainty rather than collective nationalist narratives.

This study is primarily grounded in postcolonial theory, which provides the central lens for examining colonial power, resistance, and identity formation. Trauma and memory studies are employed as complementary frameworks to analyze how historical violence is narrated and transmitted across generations, while narrative ethics informs the discussion of responsibility, witnessing, and moral ambiguity in literary representation. These approaches are not treated as competing paradigms but as intersecting tools that illuminate different dimensions of postcolonial experience. Postcolonial theory functions as the primary interpretive lens, while trauma studies and narrative ethics are mobilized as supplementary frameworks to illuminate psychological, ethical, and representational dimensions rather than to override historical analysis.

## Historical trauma and the politics of memory

When Ngugi wa Thiong’o and Abdulrazak Gurnah write about colonial legacies, they are not simply telling stories—they are excavating wounds that still shape East African societies today. Historical trauma runs through their novels not as abstract background but as a living force that determines how characters speak, identify themselves, and make ethical choices. This trauma—from the violence of conquest to the tearing apart of communities to the forced adoption of foreign values—inhabits both individual minds and shared cultural memory. As Frantz Fanon powerfully observed, “decolonization is always a violent event” that leaves deep psychological wounds which “the apotheosis of independence” alone cannot heal ( 1963, 1, 35). For both writers, confronting this trauma through literature becomes both a political act of resistance and an aesthetic challenge: how to bear witness to suppressed histories while creating art that does not simply reproduce colonial violence.

This section focuses specifically on how trauma shapes narrative structure and the politics of silence in each author — a question distinct from the collective and intergenerational dimensions examined in subsequent sections. Ngugi’s *A Grain of Wheat*, written during Kenya’s independence, captures this challenge through its fragmented narrative structure. The central character Mugo, tormented by guilt for betraying the freedom fighter Kihika, embodies the moral ambiguities of colonial resistance. When Mugo publicly confesses, “Kihika came to me by night. He put his life into my hands, and I sold it to the whiteman. And this thing has eaten into my life all these years,” Ngugi exposes how guilt and secrecy permeate the personal and collective memories of liberation. This moment reveals how the past continues to haunt the present, disrupting simplistic narratives of heroism or betrayal ( Thiong’o 1967, 187). The novel’s shifting timelines and multiple perspectives mirror the psychological disorientation of a society emerging from colonial domination-where memory itself becomes contested territory. As Abdul R. JanMohamed observes, Ngugi’s novels confront the crisis of postcolonial identity by grappling “with difficulties of cultural reintegration and regeneration in the wake of an anticolonial war” ( 1983, 10). Ngugi’s narratives not only illustrate the struggles of cultural reintegration in a postcolonial context but also reflect the deep-seated tensions between modernity and tradition, as characters navigate their identities amidst the remnants of colonial influence and the quest for authentic self-expression. This exploration highlights the complexities of navigating cultural identity in the wake of colonialism, where individuals must reconcile their heritage with the influences of a changing world. The narratives often showcase the challenges of reclaiming cultural roots while engaging with modern societal structures, creating a dynamic interplay of past and present, tradition and innovation.

Ngugi’s treatment of language politics in *A Grain of Wheat* extends beyond the opposition between colonial and indigenous languages to include silence itself as a fraught mode of communication. Mugo’s withdrawal from speech is repeatedly misread by others as moral depth and political integrity, when in fact it masks fear, guilt, and a desire for isolation. As critics have observed, the novel stages a tragic irony in which “Mugo, an outsider whose silence and inarticulateness is taken for depth and courage,” becomes the figure chosen by others as “confidante and confessor,” even though his “dearest wish … is to be left alone” (1967, 10). This misrecognition reveals how silence can function as a form of false authority, invested with ethical meaning by the community while remaining internally hollow. From the perspective of language politics, Ngugi thus exposes silence not as an innocent alternative to speech but as a politically consequential position—one that enables evasion of responsibility while being mistakenly read as resistance. The novel ultimately insists that ethical engagement requires articulation, however painful, and that silence under colonial and postcolonial conditions can reproduce harm rather than transcend it.

By contrast, *Petals of Blood* traces silence back to a more primal, traumatic origin. In the childhood episode where the boy is lost in the forest, silence is experienced not as chosen withdrawal but as existential terror: a “total silence made more silent by the noises of insects and birds,” a world “without human voices” ( 1977, 243). This moment figures silence as a form of near-erasure, a state in which the absence of language threatens psychic annihilation rather than offering refuge. Read together, these scenes suggest that Ngugi understands silence not as neutral but as historically produced: what begins as traumatic isolation in *Petals of Blood* reappears in *A Grain of Wheat* as a politically consequential refusal to speak. Language politics in Ngugi’s work thus encompass not only the struggle between colonial and indigenous languages but also the ethical demand to move from silence toward articulation as a condition of communal responsibility.

Gurnah’s *By the Sea* offers a strikingly different politics of silence, shaped not by communal misrecognition or primal trauma but by bureaucratic surveillance and linguistic vulnerability. During his interrogation as an asylum seeker, Saleh Omar reflects that he “knew the meaning of silence, the danger of words,” choosing to withhold speech while articulating a rich, ironic interior monologue that exposes Europe’s moral hypocrisy ( 2001, 12). Unlike Mugo’s silence in *A Grain of Wheat*, which enables ethical evasion, Saleh’s silence is an act of self-preservation under conditions where speech is subject to judgment, suspicion, and institutional control. Nor does this silence resemble the terror of voicelessness in *Petals of Blood*; rather, it is a conscious strategy shaped by an acute awareness of power asymmetry. Gurnah thus reframes silence not as moral failure or traumatic absence, but as an ethical response to coercive listening regimes, where language functions less as a medium of truth than as a test of credibility. In this context, silence becomes a form of narrative agency—an inward space where critique survives even when speech would invite punishment.

Gurnah takes a different approach in *Afterlives* ( 2020), which explores the colonial and postcolonial legacy of German East Africa through characters scarred by displacement, servitude, and war. Through the figure of Hamza, a former askari (colonial soldier), the novel confronts the persistent aftershocks of trauma. Hamza reflects on his fragmented past, remarking: “You want me to tell you about myself as if I have a complete story but all I have are fragments which are snagged by troubling gaps, things I would have asked about if I could, moments that ended too soon or were inconclusive” ( *Afterlives*, 2020, 157). This admission emphasizes how colonial violence shapes memory and identity not through singular events but through absences and silences. As Aminatta Forna notes in her review, “Gurnah doesn’t just tell stories of empire, he dismantles its underlying assumptions,” exposing the persistent effects of colonialism on identity and power structures in postcolonial societies ( 2023).

Both authors engage with what scholar Marianne Hirsch calls “postmemory”—how trauma is transmitted to generations who did not directly experience it but inherit its emotional aftereffects ( Hirsch 2012, 5). In Ngugi’s *Petals of Blood*, characters’ nostalgia for the Mau Mau rebellion is tinged with bitterness at how independence betrayed revolutionary ideals. For them, remembering becomes a form of critique rather than consolation. The character Karega reflects bitterly that “independence had brought no relief to their suffering-only more chains, this time fashioned by their own people” ( Thiong’o 1977, 143). Through this disillusionment, Ngugi contests what Ranger has called “patriotic history”-the sanitized national narratives that erase inconvenient truths about postcolonial governance ( 2004, 218). By critiquing these narratives, Ngugi calls for a more honest examination of the socio-political realities faced by marginalized communities in the aftermath of independence.

Gurnah, meanwhile, approaches memory with more ambivalence. In *By the Sea*, the exiled protagonist Saleh Omar carries memories not as sources of empowerment but as burdens that alienate him from both host society and self. “I began to tell the story not because I wanted to be free of it,” he says, “but because I had to carry it” ( Gurnah 2021a, 207). This distinction highlights Gurnah’s narrative ethic: memory is not about closure but about responsibility-to oneself, to the past, and to those silenced by history. Senayon Olaoluwa argues that “Gurnah’s fiction presents memory as inherently problematic-neither fully redemptive nor entirely destructive, but necessary for ethical being” ( 2017, 93). Ultimately, this complexity invites readers to grapple with their own relationships to memory, urging a deeper understanding of how personal and collective histories shape identity and ethical responsibility in a postcolonial context.

What both writers reject is the idea that historical trauma can be neatly represented or resolved. Instead, they use fragmentation, multiple perspectives, and nonlinear chronology to mirror the disorientation that trauma creates. Their work reflects what trauma theorist Cathy Caruth describes as the “unclaimed experience”-events experienced too suddenly to be fully processed, which return insistently in narrative form ( 1996, 4). Literature becomes a medium through which these unassimilated experiences can be revisited-not for recovery, but for ethical remembrance.

In *Afterlives*, Abdulrazak Gurnah portrays a brutal episode in which the German colonial officer Feldwebel Walther accuses Hamza of betrayal and strikes him violently with a weapon, causing a deep wound to his hip and rendering him unconscious ( 2020, 118). This moment of colonial violence is not depicted as an isolated act of cruelty but rather as part of a broader system in which domination over colonized bodies is both routine and instrumental. Gurnah’s narrative emphasizes how trauma is registered through the physical body, illustrating the colonial enterprise’s reduction of human life to a controllable and punishable surface. This aligns with Elaine Scarry’s argument that “the body in pain” becomes the site through which power is materialized and contested ( 1985, 27).

Moreover, both authors directly challenge official colonial archives that silenced the colonized. They recover alternative histories-oral traditions, family stories, testimonies of resistance-while acknowledging that remembering is also an act of narration, subject to its own distortions and gaps. As Spivak reminds us, the postcolonial writer must engage in a “strategic use of positivist essentialism” that recognizes the constructed nature of all historical narratives while still insisting on the political necessity of certain counter-narratives ( 1988, 13). By inscribing trauma into their texts’ very structure, Ngugi and Gurnah perform a radical act of political memory-one that resists erasure and insists on the ethical importance of bearing witness, even when complete understanding remains elusive.

It’s worth noting that both writers are responding to specific historical contexts that shaped their literary imaginations. Ngugi’s formative experiences during Kenya’s State of Emergency (1952-1960) and the violent suppression of the Mau Mau rebellion inform his representation of trauma as explicitly political and demanding collective response. As he remarked in an interview, “When people have gone through traumatic experiences, especially collectively, they need to remember. They need to remember the good things, but they also need to remember the bad times, because it’s a kind of collective therapy” ( Thiong’o 2006a, 22).

Postcolonial identity in both Ngugi and Gurnah is therefore sustained not through triumphalist recovery but through remembrance itself. As I have argued elsewhere, survival—both personal and cultural—depends on memory, since the subject “survives as long as [their] story is remembered,” a condition that applies as forcefully to East African postcolonial narratives as to earlier transcultural forms of resistance ( Jani 2021).

Gurnah’s perspective is shaped by his experience of exile following Zanzibar’s 1964 revolution and the subsequent ethnic persecution of Arab minorities. This personal history perhaps informs his more ambivalent approach to memory-his awareness of how even resistance movements can reproduce violence and exclusion. As he noted in his Nobel lecture, “I came to understand that this was the afterlife of colonialism, when difference could mean expulsion or prison or worse” ( Gurnah 2021b). This biographical context does not determine their literary choices but does illuminate how personal history shapes aesthetic and ethical vision.

Through their divergent but complementary approaches, both writers demonstrate that addressing historical trauma requires not just political change but new forms of narrative that can accommodate complexity without reducing it to simple morality tales. Their work suggests that the past is never simply past but continues to structure the present-demanding not resolution but ongoing ethical engagement.

## Colonialism, collective trauma, and the burden of memory

Whereas the previous section examined how trauma shapes individual narrative voice and the politics of silence, this section addresses a distinct question: how colonialism operated as a collective assault on communal identity and cultural continuity, and how both authors represent the burden this places on communities across time. Unlike individual trauma, collective trauma affects a group’s sense of identity and continuity (See Jani 2020, 4). Both Ngugi and Gurnah explore this dimension of colonial experience, though they frame the “burden of memory” in distinctly different ways.

Ngugi’s *The River Between* dramatizes how colonialism fractures indigenous communities from within. Set during early British colonization in Kenya, the novel follows Waiyaki’s failed attempt to reconcile Christian converts and traditionalists in his divided community. Waiyaki, a young Gikuyu man, is believed by his father, Chege, to be the prophesied savior who will deliver the Gikuyu from colonial domination. Acting on this belief, Chege sends his son to study with white missionaries to acquire Western knowledge—warning him, however, not to be corrupted by the colonizers’ culture and vices. The tragedy emerges not just from external colonial pressure but from internal ruptures—showing how even resistance to colonialism often led to new forms of division and betrayal. What’s remarkable about Ngugi’s approach is that he does not present this trauma as simple victimhood, but as a complex process where colonized people make difficult choices within severely constrained circumstances. As Oliver Lovesey argues, “Ngugi’s early fiction articulates a complex vision of pre-colonial Gikuyu culture as neither utopian nor static, but as dynamic and containing its own contradictions” ( 2000, 28). This complex portrayal not only questions the idea of a single response to colonialism but also highlights the strength of communities as they confront the challenging issues of cultural identity and moral choices during a time of significant change.

Ngugi’s *The River Between* explores the psychological conflict faced by colonized subjects navigating competing systems of value. Consider how Ngugi describes Waiyaki’s dilemma: “He was being crushed by everybody. In school, in church, they told you: trust in the Lord. But the Lord never spoke against robbing people of their land” ( Thiong’o 1965, 118). This internal conflict-between accepting colonial institutions that might provide practical tools for survival versus rejecting them to preserve cultural integrity-creates a psychological double-bind that many characters cannot resolve. The collective trauma lies precisely in this impossible choice. “The colonial situation created a peculiar form of psychological violence,” Emmanuel Obiechina argues, adding, “the violence of having to choose between incompatible systems of meaning and value,” a condition that defines the fractured inner world of Ngugi’s characters and the broader experience of colonized societies ( Obiechina 1990, 76).

Gurnah’s approach to collective trauma differs crucially from Ngugi’s communal fracture model: rather than depicting communities divided from within, Gurnah focuses on how colonial systems erased social bonds across generations and how this erasure is compounded by bureaucratic indifference in the postcolonial present. In *Afterlives*, the character Ilyas, indoctrinated into the German military, returns to his community alienated and violent, showing how colonial systems deliberately broke social bonds. What makes Gurnah’s portrayal particularly sophisticated is his attention to how trauma lingers even in periods of apparent calm. In one haunting scene, the character Khalifa finds himself unable to walk past the former German administrative building without feeling physical distress-his body remembering what his mind cannot fully articulate. Sam Durrant suggests, “Colonial trauma lives in the body even when it escapes mental representation-creating what might be called a somatic archive of historical violence” ( Durrant 2004, 67). In this way, *Afterlives* highlights how the aftershocks of colonial violence persist long after the formal end of empire, embedded in both memory and flesh.

For both writers, memory functions as both preservative and burden. Drawing on Paul Ricoeur’s insights, we see how memory sustains identity while simultaneously perpetuating grief, guilt, and trauma ( 2004, 21). In *A Grain of Wheat*, Ngugi uses the communal preparation for Uhuru Day (Kenyan independence) to reveal how national celebration conceals unresolved personal and collective wounds. The betrayals, compromises, and silences that enabled survival during colonial rule do not simply disappear with formal independence-they become embedded in the new nation’s foundation. Gikandi observes that “Ngũgĩ’s novel destabilizes the triumphalist narrative of national liberation by revealing the hidden costs of survival under colonial rule” ( 2000, 138). This suggests that liberation, while symbolically powerful, is shadowed by the persistent weight of unhealed historical trauma.

Gurnah approaches collective trauma through experiences of dislocation and fragmentation. In *By the Sea*, Saleh Omar’s memories of Zanzibar’s colonial and revolutionary past are rendered suspect by Britain’s asylum system, which demands clear narratives and documentary evidence. This bureaucratic erasure compounds his trauma, transforming memory into another form of exile. The burden lies in having experiences that cannot be properly witnessed or validated in the new country-creating what cultural theorist Homi Bhabha might call an “unhomely” condition where one belongs neither here nor there ( Bhabha 1994, 9).

What distinguishes these authors’ approaches to collective memory is how they position their characters in relation to larger historical forces. Ngugi often frames memory as potentially redemptive if properly mobilized for political action. In *Matigari*, the titular character’s quest to reclaim his home becomes a metaphor for collective reclamation of precolonial knowledge and land. His memories of the liberation struggle, though painful, provide moral clarity and purpose. At one point, *Matigari* declares, “Yesterday I was called a terrorist, but today I am a patriot. Yesterday I lived in the mountains, fighting for this country, but today I have returned to enjoy the fruits of my sweat” ( Thiong’o 1987, 42). This statement reflects what Neil Lazarus calls Ngugi’s “dialectical understanding of memory”-one that acknowledges trauma while insisting on its transformative potential ( 1990, 211).

For Gurnah, memory rarely offers such clear direction. His characters must navigate ambiguous moral terrain where the lines between victim and perpetrator blur, and where the past offers few reliable guideposts for ethical action in the present. In *Paradise*, the young protagonist Yusuf is given as collateral for his father’s debt, becoming a captive in a merchant’s household. Yet his experience is neither simple exploitation nor benevolent mentorship-it’s a complex entanglement of care and control that reflects the ambiguous nature of power in pre-colonial East Africa. As Erik Falk notes, Gurnah “refuses nostalgic constructions of pre-colonial Africa as utopian,” instead revealing how “certain forms of domination predated European conquest,” complicating any easy dichotomy between pre-colonial innocence and colonial corruption ( 2001, 219).

Both authors recognize that the burden of memory extends to language itself. In *Decolonising the Mind*, Ngugi argues that colonialism “annihilates a people’s belief in their names, in their languages, in their environment, in their heritage of struggle, in their unity, in their capacities and ultimately in themselves” ( Thiong’o 1986, 3). His decision to write in Gikuyu rather than English represents a political rejection of colonial linguistic domination-a reclaiming of cultural memory through language. This radical choice reflects what Bill Ashcroft calls “the most complete form of linguistic abrogation”-a total rejection of the colonizer’s language as medium for authentic cultural expression ( 2001, 78).

Gurnah, while continuing to write in English, fills his prose with untranslated Kiswahili words and cultural references that resist full assimilation into European literary traditions. His characters often experience language as a site of both belonging and alienation, reflecting his more ambivalent stance toward the possibility of complete decolonization. “Gurnah’s strategic use of untranslated phrases creates what might be called ‘linguistic interruptions’,” Felicity Hand observes, adding, “moments where the dominance of English is challenged without being completely rejected” ( 2010, 68).

In *Desertion*, Gurnah examines the limits of linguistic expression by staging moments in which emotional intent must be carried across languages shaped by unequal competence and cultural expectation. When Martin Pearce writes to Rehana in Arabic, the act of composition itself becomes a struggle, since “a love letter in Arabic … required a command of convention and metaphor which was quite unlikely to be within Martin’s grasp of the language” ( Gurnah 2005, 96). Martin’s understanding of how such a letter should sound is shaped not by lived intimacy but by literary models drawn from Edward Lane’s translations of *One Thousand and One Nights*, whose stylized worlds of romance and fantasy offer guidance that proves only partially applicable to the social realities he inhabits. The episode shows that language here operates not as a transparent channel for feeling but as a culturally coded practice, where emotional expression is filtered through inherited stories and borrowed forms. Memory and desire in the novel are therefore mediated by literary imagination as much as by lived experience, underscoring how cross-cultural communication is shaped by the narratives one carries into it.

Through their divergent but complementary literary visions, Ngugi and Gurnah demonstrate that the burden of memory in postcolonial contexts is not simply about recalling past injustices-it’s about negotiating how those injustices continue to shape identity, community, and possibility in the present. Their work suggests that addressing collective trauma requires not just political change but also new forms of narrative that can accommodate historical complexity without reducing it to simple binaries of colonizer and colonized. “Postcolonial fiction at its best doesn’t simply invert colonial binaries,” Nana Wilson-Tagoe argues, adding, “but creates new conceptual spaces where alternative understandings become possible” ( 2006, 225).

## Intergenerational trauma and the transmission of cultural loss

Having examined how trauma shapes individual narrative (Section 1) and collective communal identity, this section addresses a third distinct dimension: how colonial wounds are transmitted across generations through family structures, educational systems, and language. The wounds of colonialism do not end with those who directly experienced colonial rule-they echo through subsequent generations in what scholars call “intergenerational trauma.” Marianne Hirsch’s concept of “postmemory” helps explain how children of trauma survivors inherit the emotional and narrative burdens of events they never personally witnessed ( Hirsch 2012, 5). Both Ngugi and Gurnah explore this transmission, showing how historical dislocation, linguistic suppression, and cultural losses pass between generations through family stories, silences, and embodied practices.

Ngugi’s *Petals of Blood* demonstrates this theme through its narrative structure. Told retrospectively through multiple voices, the novel reveals how the betrayal of independence ideals-particularly the co-optation of revolutionary goals by the new elite-creates inherited disillusionment. The younger character Karega discovers that his personal suffering connects to larger historical patterns: his brother’s death during the Emergency, his mother’s displacement from ancestral land, and his own exploitation as a worker all form links in a chain of connected injustices. Apollo Amoko notes that “Ngũgĩ portrays postcolonial education as a site where colonial values continue to be instilled in younger generations, creating a form of epistemic violence that perpetuates trauma” ( 2010, 42). What makes Ngugi’s portrayal particularly powerful is how he shows trauma transmission occurring not just within families but through educational systems, economic structures, and cultural institutions that perpetuate colonial mentalities even after formal independence.

Consider this passage where Karega realizes the connection between past and present oppression: “It was as if the history of his family was a reflection of the history of his country” ( Thiong’o 1977, 236). This moment of recognition transforms individual suffering into political consciousness-suggesting that understanding intergenerational trauma can become a catalyst for resistance rather than merely a source of pain. Thus, “For Ngũgĩ, the recognition of historical continuities between colonial and neocolonial oppression is the first step toward genuine liberation,” Ato Quayson argues ( 2000, 124).

Gurnah approaches intergenerational trauma more obliquely in novels like *Paradise* and *Desertion.* In *Paradise*, the protagonist Yusuf is given to a merchant as rehani (collateral) for his father’s debt—a transaction that reflects how colonial economic systems disrupted traditional family structures and created new forms of bondage. “Gurnah’s historical fiction reveals how colonialism worked through existing social structures, transforming rather than simply destroying them,” Frederick Cooper observes ( 2005, 189). As Yusuf journeys through East Africa with the merchant’s caravan, he witnesses multiple communities grappling with cultural dissolution in the face of colonial incursion. The trauma here is not just about specific violent events but about the gradual erosion of ways of life and systems of meaning that once sustained communities.

What distinguishes Gurnah’s approach is his attention to how colonial trauma operates differently across social positions. Sissy Helff observes that “Gurnah’s narratives refuse the comfort of clear moral positions, showing instead how colonial entanglements produced complex ethical dilemmas that continue to resonate across generations” ( 2015, 77). In *Desertion*, he traces a forbidden love story across generations, showing how British colonial presence created intimate disruptions that continue to shape family dynamics decades later. Through the character Rashid, who attempts to piece together his family’s fragmented history, Gurnah suggests that postcolonial identity formation requires confronting uncomfortable truths about how colonial relationships infiltrated the most personal aspects of life.

Both authors recognize language as a critical site of intergenerational trauma transmission. Ngugi famously describes in *Decolonising the Mind* how colonial education created a “cultural bomb” that made children ashamed of their mother tongues and ancestral cultures ( Thiong’o 1986, 3). In his fiction, characters who have internalized colonial values often speak in linguistic registers that separate them from their communities-creating what he calls a “dislocating distance” between colonized subjects and their cultural roots. His commitment to writing in Gikuyu represents a rejection of this linguistic trauma and an attempt to heal the rift between generations. As Thiong’o himself argues, “Language is central to a people’s definition of themselves and their relationship to the universe. To control people’s language is to control their tools of self-definition” ( Thiong’o 1993, 30).

Gurnah’s characters often inhabit multiple linguistic worlds, reflecting his interest in cultural hybridity rather than purity. In *By the Sea*, the protagonist Saleh uses his knowledge of language to strategically navigate systems of power-sometimes embracing linguistic ambiguity as a form of resistance. Yet Gurnah also shows how language loss creates profound intergenerational disconnections. Fawzia Mustafa observes that “Gurnah’s multilingual characters embody the complex linguistic legacies of colonialism-neither fully at home in colonial languages nor able to return to some pure pre-colonial linguistic identity,” a dynamic clearly illustrated in *Afterlives*, where the character Ilyas’s German education separates him from his sister Afiya both physically and culturally—a separation that ripples through subsequent generations in her family ( 2015, 143; Gurnah 2020, 28-50).

What both authors ultimately suggest is that addressing intergenerational trauma requires more than simply remembering the past-it demands creating new narratives that acknowledge historical wounds while imagining different futures. Gikandi argues that “Ngũgĩ’s turn to indigenous narrative forms represents an attempt to heal the rupture between traditional knowledge and modern experience” ( 2000, 195). In this context, Ngugi tends to frame this process in collective terms, emphasizing how shared storytelling can reconnect communities with precolonial knowledge systems and values. His later works like *Wizard of the Crow* employ elements of magical realism and folk traditions to create narrative frameworks that resist colonial temporal and epistemological structures ( Thiong’o 2006b).

Gurnah, by contrast, focuses more on how individuals navigate traumatic legacies through personal choices and relationships. His characters rarely find complete resolution or return to an imagined precolonial wholeness. Instead, they forge tentative connections across cultural and historical divides-suggesting that healing might come not through grand political projects but through small acts of understanding and recognition. Hedley Twidle observes that

Gurnah’s fiction offers no easy consolations or redemptive narratives, but rather a complex ethics of witnessing that acknowledges both the persistence of trauma and the possibility of limited forms of reconciliation ( 2019, 89).

In this view, narrative becomes a form of ethical engagement—one that neither erases pain nor promises closure, but offers a space where fragmented histories can be held, heard, and honored. Such an approach insists that even amid dislocation, there remains a possibility for meaning and connection.

Despite these differences, both writers demonstrate that literature itself can be a vehicle for addressing intergenerational trauma-not by “curing” it, but by creating spaces where it can be witnessed, articulated, and potentially transformed. Their work suggests that the transmission of trauma across generations is not inevitable or deterministic, but rather a complex process that can be interrupted by conscious engagement with history, language, and narrative. LaCapra argues that

Working through trauma involves the effort to articulate or rearticulate affect and representation in a manner that may never transcend, but may to some viable extent counteract, a reenactment, or acting out, of that disabling dissociation ( 2001, 42).

Literature, in this context, becomes a medium not of resolution but of ethical reckoning—offering readers a space to confront the residues of violence while imagining more humane futures.

## Language politics and narrative authority

Few aspects of postcolonial writing generate more debate than language choice-what it means to write in colonial versus indigenous languages, and how this choice shapes narrative authority and audience. Ngugi and Gurnah take dramatically different positions on this question, reflecting broader tensions within postcolonial literary practice.

In his 1986 *Decolonising the Mind*, Ngugi’s famous “farewell to English” marked a radical turning point in his career. After writing his early novels in English, he committed to writing primarily in Gikuyu-a decision explained in his influential essay collection *Decolonising the Mind.* For Ngugi, language is never neutral; it carries the worldview, values, and power structures of its cultural origins. “Language carries culture,” he writes, “and culture carries, particularly through orature and literature, the entire body of values by which we perceive ourselves and our place in the world” (1986, 16). This position aligns with what Sapir and Whorf proposed about the relationship between language and worldview-that the languages we speak fundamentally shape how we perceive reality ( 1956, 213).

This perspective emerges powerfully in novels like *Matigari*, where Ngugi employs Gikuyu oral storytelling techniques and proverbs that would be difficult to fully replicate in English. When the protagonist asks, “Where can a person wearing the belt of peace find truth and justice in this country?” the question resonates with specific cultural connotations that connect to Gikuyu ethical traditions (1987, 71). The novel’s structure-blending realistic narrative with mythic elements-draws from indigenous narrative forms rather than European literary conventions. Even in translation, this grounding in Gikuyu cultural epistemology creates a narrative authority rooted in non-Western knowledge systems. As Gikandi observes, “Ngũgĩ’s return to Gikuyu was not simply a rejection of English but an affirmation of alternative ways of knowing and narrating that had been delegitimized by colonialism,” a move that reclaims narrative sovereignty and asserts the validity of suppressed intellectual traditions ( 2000, 205).

Gurnah, by contrast, has written exclusively in English, though his prose is deliberately inflected with Kiswahili rhythms, untranslated words, and cultural references that create what literary scholar Sukhdev Sandhu calls a “thickly accented English” ( 2020). According to Chantal Zabus, “Such strategies of relexification and translingualism create a form of ‘decolonized English’ that subverts from within rather than rejecting outright” ( 2007, 142). In *Paradise*, phrases and concepts from Kiswahili, Arabic, and various East African languages create a linguistic texture that reflects the cultural hybridity of pre-colonial and colonial East Africa. This approach does not reject English so much as transform it-making the colonial language bear witness to experiences it was often used to erase.

Consider how Gurnah describes the merchant Hamid in *Paradise*: “He spoke Arabic slowly and nasally, stretching the words in a way that gave his speech gravity” ( 1994, 25). As David Callahan notes, “Gurnah’s code-switching and linguistic hybridization reflect the complex reality of coastal East African societies, where language choice has always been political” ( 2011, 119). This attention to the materiality of language-how it sounds, how it is embodied-suggests that even while writing in English, Gurnah remains deeply attentive to the multilingual reality of East African societies. His characters often move between languages strategically, using different linguistic registers depending on context and power dynamics.

The contrast between these approaches reveals different understandings of decolonization itself. For Ngugi, true decolonization requires a decisive break with colonial languages and a return to indigenous modes of expression. His position aligns with what Ghanaian philosopher Kwasi Wiredu calls “conceptual decolonization”-the effort to think through indigenous conceptual frameworks rather than imposed Western ones ( 1996, 81). Gurnah’s approach suggests that decolonization might involve transforming colonial inheritances from within-a position closer to Homi Bhabha’s concept of hybridity as a potentially subversive “third space” that disrupts binary oppositions between colonizer and colonized ( 1994, 38).

These language choices directly affect how each author constructs narrative authority. Ngugi often employs communal narrators or multiple perspectives that draw from oral storytelling traditions, where authority is distributed rather than centralized. In *Petals of Blood*, the investigation into a murder becomes an opportunity for multiple characters to tell their versions of Kenya’s post-independence history. This polyvocality challenges both colonial master narratives and the new nationalist mythologies that replaced them. Abiola Irele notes that “Ngũgĩ’s narrative strategy reflects a democratic impulse-a refusal to privilege any single voice as the bearer of definitive truth,” thereby enabling a more inclusive and contested understanding of historical memory and postcolonial identity ( 2001, 156).

Ngugi dramatizes the politics of linguistic authority most sharply in *Wizard of the Crow*, where the state’s power depends on monopolizing not only institutions but the very terms through which reality is described. In one scene, the regime’s ministers celebrate replacing inherited intellectual traditions with the Ruler’s ideological doctrine, insisting that older frameworks should be discarded: “We cannot allow the sepulchral mud of the dead to besmirch the spectacular mind of the living” ( Thiong’o 2006b). The grotesque excess of this phrasing is not decorative; it exposes how authoritarian discourse works by renaming coercion as progress and by staging the Ruler’s language as the only legitimate idiom of thought. Even apparently conciliatory speech becomes a tactic of control, as when the Ruler replies to foreign pressure with the performative compromise, “Then let’s agree to disagree” ( Thiong’o 2006b). Read closely, these moments show narrative authority emerging through satire: the novel invites readers to hear the inflation, euphemism, and theatricality of official language as evidence of its ethical emptiness, while aligning credibility with the counter-voices that resist the regime’s linguistic capture of “truth.”

Gurnah typically uses more conventional first-person or limited third-person narrators, but undermines their authority through narrative unreliability and perspectival shifts. In *Desertion*, different characters tell conflicting versions of the same events, leaving readers to navigate between partial truths. This approach reflects Gurnah’s skepticism toward totalizing narratives of any kind-whether colonial, nationalist, or traditionalist.

Gurnah’s narrative authority in *Desertion* is established through a deliberately limited focalization that repeatedly foregrounds uncertainty rather than mastery. Early in the novel, perception is presented as inference and self-correction: Frederick “assumed” the wakil’s posture came from “a lifetime of crooked clerking,” and even his descriptive language is rehearsed as something provisional—“Frederick tried the phrase to himself two or three times” until he could write it down ( Gurnah 2005, 107). The text also insists on interpretive instability in colonial contact, as Frederick admits that he “could not be sure” whether the smiles around him were “kind or otherwise” ( Gurnah 2005, 107). These small but persistent hesitations matter: they model a narrative ethic in which knowledge is partial, perception is situated, and authority is continuously undermined from within the act of telling—preparing the ground for the novel’s later perspectival shifts and contested accounts.

Gurnah’s narrators often acknowledge the limits of their understanding, as when Saleh in *By the Sea* admits: “I tell you what I know, which is only a little of what there is to tell” (2021, 97). Tina Steiner argues that “This narrative humility represents an ethical stance-a recognition that all perspectives are necessarily partial and situated,” emphasizing how Gurnah’s fiction privileges complexity over certainty, and empathy over ideological closure ( 2009, 50).

What unites these divergent approaches is a shared commitment to challenging Western epistemological authority-the assumption that European ways of knowing, narrating, and organizing experience are universal or superior. Both authors, through different strategies, assert the legitimacy of East African experiences and perspectives that colonial discourses marginalized. Their work demonstrates that language politics in postcolonial literature are not simply about which language one writes in, but about how one uses language to reconstruct authority, memory, and identity in the aftermath of colonial epistemic violence. Spivak write, “The postcolonial critic is obliged to engage in a persistent critique of ‘the effects of epistemic violence,’ while recognizing her own complicity in those very structures,”—a reminder that literary resistance must be self-reflective, ethically grounded, and attuned to the lingering traces of the systems it seeks to oppose ( 1999, 367).

## Exile, return, and the geography of identity

While both Ngugi and Gurnah have experienced forced exile from their homelands, they incorporate this experience into their fiction in markedly different ways. For Ngugi, exile functions primarily as a political condition to be overcome through commitment to collective liberation, while for Gurnah, it becomes a permanent existential state that fundamentally reshapes identity.

Ngugi’s experience of detention without trial by the Kenyan government in 1977 and subsequent exile profoundly influenced works like *Devil on the Cross*, which he famously wrote on toilet paper while imprisoned ( Thiong’o 1980). After his release, continued harassment forced him to leave Kenya, yet his fiction maintains an unwavering commitment to the specific geography of his homeland-particularly the Gikuyu highlands. Even when writing from abroad, his narrative imagination remains firmly rooted in Kenyan soil, villages, and political struggles. “For Ngũgĩ, exile is a temporary condition to be endured but ultimately overcome through political transformation,” Gikandi observes, emphasizing how Ngugi’s diasporic position does not diminish his national literary mission ( 2000, 197).

In *Matigari*, the protagonist returns from years of fighting in the liberation struggle to find that independence has failed to deliver genuine freedom. His journey through the post-independence landscape becomes a searing critique of neocolonial corruption. Yet despite this disillusionment, the novel maintains faith in eventual collective redemption. When *Matigari* buries his weapons rather than using them, declaring “I shall return,” he affirms the possibility of genuine homecoming-not just physical return but the restoration of a just social order (1987, 175). “Ngũgĩ’s vision of return is both literal and metaphorical-a physical homecoming and a restoration of ethical social relations,” Lazarus argues, highlighting how political hope in *Matigari* is grounded in moral regeneration as much as in spatial belonging ( 1990, 217).

Gurnah’s approach to exile reflects his own experience fleeing Zanzibar’s revolutionary violence as a teenager-a departure that preceded decades before he could safely return. His characters rarely experience straightforward homecomings. In *By the Sea*, Saleh Omar arrives in England as an elderly asylum seeker, carrying only a mahogany box and fragments of his past. Unlike Ngugi’s politically engaged exiles, Saleh’s displacement is marked by profound ambivalence: “I did not leave my country for political reasons. I left because there was nothing else I could do” (Gurnah 2021, 4). This statement captures a crucial difference between the authors: while Ngugi frames exile primarily through political oppression that can potentially be overcome, Gurnah explores how exile becomes embedded in identity itself-creating what Edward Said called “a discontinuous state of being” ( 2000, 177).

The geography of identity in these authors’ works reflects their different relationships to nationalist politics. Ngugi’s fiction, even at its most critical, maintains faith in the concept of national liberation-the idea that colonial borders, though artificial, can become meaningful containers for collective self-determination. His detailed descriptions of Kenyan landscapes are not merely aesthetic but political: they reassert indigenous connections to land that colonialism attempted to sever. As Patrick William argues, “Ngugi’s topographical specificity-his naming of hills, rivers, and villages-performs a kind of textual reclamation of territory,” underscoring how landscape in his fiction becomes a site of resistance and cultural restoration ( 2004, 89).

Gurnah, by contrast, often depicts East Africa as it existed before modern nationalism-a coast shaped by centuries of trade, cultural exchange, and movement. In *Paradise*, the caravans that cross multiple territories reflect a precolonial geography defined by networks rather than borders. Falk observes that “Gurnah’s fiction presents identity as inherently relational and contingent-shaped by movements between places rather than fixed attachment to any single location,” an idea that underpins Gurnah’s reconstruction of precolonial East Africa as a space of mobility and fluid affiliations ( 2007, 241). This historical perspective allows Gurnah to critique both colonial divisions and the nationalist responses that sometimes reinforced them. For his characters, identity emerges not from rootedness in a single place but from navigation between multiple cultural, linguistic, and geographical affiliations.

Both authors recognize the body itself as a site where exile and belonging are negotiated. In Ngugi’s *Wizard of the Crow*, the dictator’s mysterious illness-a body that bloats uncontrollably-becomes a metaphor for the corruption of postcolonial leadership. The protagonist’s healing practices, drawing from indigenous knowledge, suggest that physical and political recovery require reconnection with suppressed traditions. “Ngugi uses bodily metaphors to express how political corruption manifests as a physical and spiritual disease requiring indigenous remedies,” Gikandi notes, emphasizing the novel’s vision of healing as both a cultural and corporeal imperative ( 2006, 168).

Gurnah’s characters often bear physical marks of displacement-scars, illnesses, or aging that track their journeys through hostile environments. In *Afterlives*, Hamza’s body carries a scar that symbolizes how colonial violence becomes physically incorporated into the colonized subject. Yet Hamza’s eventual intimate relationship with Afiya suggests that even these bodily wounds can become sites of tentative healing through human connection. As Emma Hunt observes, “For Gurnah, the body retains traces of historical violence but also possesses the capacity for new forms of sensuous connection that might partially redeem past trauma,” suggesting that healing, while incomplete, remains possible through affective intimacy ( 2014, 104).

What emerges from this comparison is a recognition that exile in postcolonial literature functions not just as biographical background but as a fundamental condition that shapes narrative form, characterization, and ethical vision. As JanMohamed argues, “For Ngugi, exile heightens rather than diminishes political commitment-creating what might be called a ‘specular border intellectual’ whose distance enables clearer critique,” reinforcing how displacement in Ngugi’s work sharpens, rather than obscures, the vision for collective liberation ( 1992, 97). Therefore, Ngugi’s approach emphasizes how exile intensifies political commitment and clarifies the imperative for collective liberation. His characters may be displaced, but their moral compass remains oriented toward homeland and community.

Gurnah’s vision suggests that exile-once experienced-permanently alters one’s relationship to all places and identities. He “transforms exile from mere displacement into a complex ethical stance-a position from which to question the naturalized categories that enable exclusion,” Steiner stresses ( 2015, 138). Therefore, Gurnah’s characters rarely find complete belonging anywhere, yet this very unbelonging becomes a source of insight. As Saleh reflects in *By the Sea*: “I know what it is like to be an outsider, to be the one left out of account” (Gurnah 2021, 142). This perspective enables a particular kind of ethical imagination-one that recognizes the contingency of all social arrangements and the fragility of human connection across difference.

Despite these contrasts, both authors ultimately reject simplistic divisions between exile and belonging, suggesting instead that identity in postcolonial contexts involves continuous negotiation between displacement and attachment, between the specific wounds of history and the universal human need for home. As Salman Rushdie famously wrote about exile writers, they “are haunted by some sense of loss, some urge to reclaim, to look back, even at the risk of being mutated into pillars of salt” ( Rushdie 1991, 10). Therefore, we can say that both Ngugi and Gurnah look back, but their gazes serve different purposes-Ngugi to mobilize recovery, Gurnah to witness complexity.

## Conclusion

This comparative analysis of Ngugi wa Thiong’o and Abdulrazak Gurnah has demonstrated that these authors represent two distinct yet complementary ethical visions of how postcolonial literature might address colonial legacies. Ngugi embraces clear political commitment and collective mobilisation, foregrounding linguistic decolonisation as the basis for cultural reclamation. Gurnah explores ambiguity and individual psychological complexity, presenting exile as a permanent condition and moral agency as something preserved within, rather than against, historical constraint.

Across the four themes examined — historical trauma, collective memory, intergenerational transmission, and language politics — the analysis has shown that their differences are not merely stylistic but philosophical, reflecting divergent understandings of what decolonisation requires and what literature can ethically offer. What unites them is a shared commitment to narrative justice: the belief that telling stories can contest historical erasure and create space for more ethical relationships between past, present, and future.

This study contributes to postcolonial literary studies by demonstrating the vital importance of East African literature to broader theoretical conversations in the field, cautioning against treating “African literature” as a monolithic category. As Gikandi argues, “the task of postcolonial criticism is not to choose between competing modes of resistance but to recognise their distinct contributions to decolonising knowledge and imagination” ( 2001, 640). In this spirit, Ngugi’s political clarity and Gurnah’s sophisticated ambiguity are best understood as complementary strategies for addressing different dimensions of postcolonial experience.

## Informed consent was not obtained/applicable

The study does not involve human participants or their data, simply state so.

## AI use

During the preparation of this work the author used ChatGPT TOOL, version 40, in order to improve language and readability. After using this tool, the author reviewed and edited the content as needed and takes full responsibility for the content of the publication.

## Data Availability

The Zenodo repository for “Reimagining Identity in Postcolonial East African Literature”, DOI: 10.5281/zenodo.16743946 ( https://doi.org/10.5281/zenodo.16743946) This project contains the following underlying data:
- References.docx (List of bibliographic references used in the manuscript). Data are available under the terms of the Creative Commons Attribution 4.0 International license (CC-BY 4.0).
