# Supplementary material for: Reimagining Identity in Postcolonial East African Literature: A Comparative Analysis of Ngugi wa Thiong'o and Abdulrazak Gurnah
Source: F1000Res. 2026 Mar 19;14:1025. Originally published 2025 Oct 2. [Version 4] doi: 10.12688/f1000research.167352.4 (PMC12966795; doi:10.12688/f1000research.167352.4)
Supplement: Peer review history

# Reviewer report: Reviewer response for version 3

**DOI:** 10.5256/f1000research.195731.r461526

**Published:** 2026-03-06

**Authors:** Kristiawan Indriyanto

**Affiliations:** 1 Universitas Prima Indonesia, Medan, Indonesia

Regarding this article, i have several concerns to bear.

 First of all, I think the abstract should be around 200 words at minimum, i don't see the justification for comparing these two authors as a comparative study. Moreover, you should more explicitly stated the similarities/differences of each author, right now it still sounds too general/vague.

 In my opinion, some parts of your findings/historical circumstances are overlapping quote a bit

- Historical trauma and the politics of memory
- Colonialism, collective trauma, and the burden of memory
- Intergenerational trauma and the transmission of cultural loss

you might consider take a closer look on these parts and ensure there are no overlapping/repeated argument, a clearer signposting between section would be appreciated.

 Isn't your conclusion is a bit long? I think some parts are trying to introduce a new topic/area of analysis in which a conclusion should not do. Just briefly restate your argument, make sure there is no new ideas/quotation being stated.

 Avoid also too personal opinion, i.e. "I’m struck by how both authors"

Is the work clearly and accurately presented and does it cite the current literature?

Yes

If applicable, is the statistical analysis and its interpretation appropriate?

Not applicable

Are all the source data underlying the results available to ensure full reproducibility?

No source data required

Is the study design appropriate and is the work technically sound?

Yes

Are the conclusions drawn adequately supported by the results?

Yes

Are sufficient details of methods and analysis provided to allow replication by others?

Yes

Reviewer Expertise:

Postcolonial literature; Indigeneity ; Ecocriticism ; Narrative Theory

I confirm that I have read this submission and believe that I have an appropriate level of expertise to confirm that it is of an acceptable scientific standard.

# Response

**Published:** 2026-03-09

**Authors:** Bushra Juhi Jani

**Affiliations:** Supporting Sciences Unit, Al-Nahrain University, Baghdad, Baghdad Governorate, Iraq

Dear Reviewer,

 Thank you sincerely for your careful reading of the manuscript and for your constructive and detailed feedback. I have taken all your comments seriously and have revised the manuscript accordingly. Below I address each point in turn.

 1. Abstract length

 I agree that the abstract was insufficiently developed. I have expanded it to approximately 200 words, ensuring it clearly summarises the paper's argument, methodology, and key findings.

 2. Justification for the comparative study

 I have added a more explicit rationale for comparing Ngugi wa Thiong'o and Abdulrazak Gurnah, foregrounding their shared postcolonial East African context while highlighting the distinct national, linguistic, and diasporic positions from which each author writes. The revised introduction makes the comparative logic clearer and more compelling.

 3. Similarities and differences stated more explicitly

 I have restructured the analysis to ensure that the similarities and differences between the two authors are stated explicitly rather than implied.

 4. Overlapping sections

 I have carefully reviewed the three sections you identified:

 - Historical trauma and the politics of memory

 - Colonialism, collective trauma, and the burden of memory

 - Intergenerational trauma and the transmission of cultural loss

 I have reorganised and distinguished these sections more clearly, removing repeated arguments and ensuring each section makes a distinct contribution.

 5. Conclusion

 I have substantially revised the conclusion. All new ideas, new quotations, and new areas of analysis have been removed. The conclusion now briefly restates the paper's central argument and summarises its main contributions without introducing new material.

 6. Personal opinion

 The phrase "I'm struck by how both authors" and any similarly personal formulations have been removed and replaced with appropriately academic language.

 I am grateful for the time and expertise you brought to this review. I believe the revised manuscript is significantly stronger as a result of your comments.

 Yours sincerely,

 Bushra

# Reviewer report: Reviewer response for version 3

**DOI:** 10.5256/f1000research.195731.r454120

**Published:** 2026-02-03

**Authors:** Thomas A Mattappallil

**Affiliations:** 1 Rajagiri College of Social Sciences, Kochi, Kerala, India

The author has incorporated changes.

Is the work clearly and accurately presented and does it cite the current literature?

Yes

If applicable, is the statistical analysis and its interpretation appropriate?

Yes

Are all the source data underlying the results available to ensure full reproducibility?

Yes

Is the study design appropriate and is the work technically sound?

Yes

Are the conclusions drawn adequately supported by the results?

Yes

Are sufficient details of methods and analysis provided to allow replication by others?

Yes

Reviewer Expertise:

NA

I confirm that I have read this submission and believe that I have an appropriate level of expertise to confirm that it is of an acceptable scientific standard.

# Reviewer report: Reviewer response for version 2

**DOI:** 10.5256/f1000research.194880.r450623

**Published:** 2026-01-20

**Authors:** Thomas A Mattappallil

**Affiliations:** 1 Rajagiri College of Social Sciences, Kochi, Kerala, India

If applicable, is the statistical analysis and its interpretation appropriate?

 the above question is not relevant to this study discussed in the article since, it is purely a qualitative study.       

 The author has placed its idea and logically, coherently following its methodology. The work is an outstanding example of research and methodological practice that is particularly well suited for teaching, as it presents key concepts in a clear, well-structured, and accessible manner without sacrificing analytical depth. It effectively demonstrates the application of theory to practice through a transparent and coherent methodology, supported by illustrative examples that encourage critical engagement from students at both undergraduate and postgraduate levels. In addition, the study has generated high-quality data and/or resources that are rigorously produced, well-documented, and openly available, allowing for verification, reuse, and extension by other researchers. The robustness, openness, and reusability of these data and resources significantly enhance the study’s value for future research and interdisciplinary scholarship.

Is the work clearly and accurately presented and does it cite the current literature?

Yes

If applicable, is the statistical analysis and its interpretation appropriate?

Yes

Are all the source data underlying the results available to ensure full reproducibility?

Yes

Is the study design appropriate and is the work technically sound?

Yes

Are the conclusions drawn adequately supported by the results?

Yes

Are sufficient details of methods and analysis provided to allow replication by others?

Yes

Reviewer Expertise:

NA

I confirm that I have read this submission and believe that I have an appropriate level of expertise to confirm that it is of an acceptable scientific standard.

# Response

**Published:** 2026-01-23

**Authors:** Bushra Juhi Jani

**Affiliations:** Supporting Sciences Unit, Al-Nahrain University, Baghdad, Baghdad Governorate, Iraq

I am sincerely grateful to the reviewer for this generous and thoughtful assessment of the revised manuscript. I appreciate the clear recognition that the study is qualitative in nature and that statistical analysis is therefore not applicable to its design or aims.

 I am particularly thankful for the reviewer’s detailed comments regarding the coherence of the methodology, the clarity of presentation, and the study’s value as a teaching and research resource. The acknowledgement of the work’s transparency, theoretical–practical integration, and the openness and reusability of its data and resources is especially encouraging, as these were central objectives of the study.

 I am also grateful for the confirmation that the study design, methodological rigor, use of current literature, and support for the conclusions are appropriate and sound. I appreciate the reviewer’s careful reading and their affirmation that the work meets a high scholarly standard.

 Thank you again for this constructive and affirming review.

# Reviewer report: Reviewer response for version 1

**DOI:** 10.5256/f1000research.184459.r435437

**Published:** 2025-12-29

**Authors:** Emmanuel Mensah Bonsu

**Affiliations:** 1 University of Cape Coast,, Cape Coast,, Ghana

Thank you for the opportunity to review this comparative analysis of Ngugi wa Thiong'o and Abdulrazak Gurnah. The paper tackles an important topic and demonstrates extensive engagement with both primary texts and secondary literature. The author demonstrates impressive familiarity with both authors' oeuvres and relevant secondary scholarship spanning postcolonial studies, trauma theory, and African literary criticism. The paper effectively situates both authors within their specific historical and biographical contexts. The thematic organization is effective, and your engagement with both primary and secondary sources demonstrates strong scholarly expertise. Despite this importance, I invite the author to consider the following suggestions.

  

1. While it generally acceptable that literary analysis should not have a clear methodology section, I would recommend that the author add a brief explanation in the introduction about the analytical approach and text selection rationale. More importantly, the author should provide summaries of each of the novels to help readers understand the text much better.
2. While page 3 states a central question, the paper’s original contribution to scholarship remains unclear. The introduction mentions that “few studies have placed them in direct conversation”, but you should articulate what new insights your analysis provides beyond existing scholarship. It will also be important to review some existing comparative studies to ground your claims.
3. Several sections (particularly on language politics and exile) rely heavily on secondary interpretations rather than direct textual analysis. Please, ensure each major claim is supported by direct textual evidence from the primary works, not just scholarly commentary about them.
4. The paper draws eclectically from multiple theoretical traditions (postcolonial theory, trauma studies, memory studies, narrative ethics) without clearly articulating how these frameworks interact or which takes precedence when they might conflict. Add a brief theoretical framework subsection clarifying how you synthesize these different approaches.
5. The conclusion could strengthen its case for why this comparison matters *now*. How does this analysis contribute to current debates in postcolonial studies? What does it offer readers?

Is the work clearly and accurately presented and does it cite the current literature?

Yes

If applicable, is the statistical analysis and its interpretation appropriate?

Not applicable

Are all the source data underlying the results available to ensure full reproducibility?

No source data required

Is the study design appropriate and is the work technically sound?

Yes

Are the conclusions drawn adequately supported by the results?

Yes

Are sufficient details of methods and analysis provided to allow replication by others?

Yes

Reviewer Expertise:

NA

I confirm that I have read this submission and believe that I have an appropriate level of expertise to confirm that it is of an acceptable scientific standard, however I have significant reservations, as outlined above.

# Response

**Published:** 2026-01-05

**Authors:** Bushra Juhi Jani

**Affiliations:** Supporting Sciences Unit, Al-Nahrain University, Baghdad, Baghdad Governorate, Iraq

We sincerely thank the reviewer for their careful reading of the manuscript and for their constructive and insightful comments. We are grateful for the positive evaluation of the paper’s scholarly engagement, thematic organization, and contribution to postcolonial African literary studies. We have revised the manuscript accordingly and believe that these changes have strengthened both its clarity and its contribution. Our responses to the reviewer’s specific comments are detailed below.

**Reviewer comment 1:**

*While it is generally acceptable that literary analysis should not have a clear methodology section, I would recommend that the author add a brief explanation in the introduction about the analytical approach and text selection rationale. More importantly, the author should provide summaries of each of the novels to help readers understand the text much better.*

**Response:**

 We thank the reviewer for this helpful suggestion. In Version 2, we have added a clear explanation of the analytical approach and text selection rationale in the introduction. The revised manuscript now explicitly states that the study adopts a qualitative comparative literary analysis grounded in close textual reading, and it explains why the selected novels represent key phases in each author’s engagement with colonial and postcolonial history.

 In addition, we have incorporated contextualized summaries of the primary novels throughout the analytical sections. Rather than isolating plot summaries into a separate section, summaries are now embedded where they are analytically relevant, allowing readers unfamiliar with the texts to follow the discussion while preserving the essay’s critical focus.

**Reviewer comment 2:**

*While page 3 states a central question, the paper’s original contribution to scholarship remains unclear. The introduction mentions that “few studies have placed them in direct conversation,” but you should articulate what new insights your analysis provides beyond existing scholarship. It will also be important to review some existing comparative studies to ground your claims.*

**Response:**

 We appreciate this important observation. In response, we have substantially revised the introduction to clarify the article’s original contribution. The revised text now explicitly articulates how the paper advances scholarship by placing Ngugi wa Thiong’o and Abdulrazak Gurnah in sustained comparative dialogue through the lenses of postcolonial ethics, trauma, memory, language politics, and narrative authority.

 We emphasize that the article contributes new insights by identifying the authors as representing contrasting yet complementary ethical models of postcolonial literary practice—one oriented toward collective political clarity and linguistic decolonization, the other toward narrative ambiguity and ethical witnessing. We have also strengthened engagement with existing scholarship to situate this contribution more clearly within current debates.

**Reviewer comment 3:**

*Several sections (particularly on language politics and exile) rely heavily on secondary interpretations rather than direct textual analysis. Please ensure each major claim is supported by direct textual evidence from the primary works.*

**Response:**

 We thank the reviewer for this valuable guidance. In Version 2, we have revised these sections to include more extensive close readings and direct quotations from the primary texts, including *A Grain of Wheat*, *Petals of Blood*, *By the Sea*, and *Afterlives*. Each major interpretive claim is now explicitly grounded in textual evidence, with secondary scholarship used to support rather than substitute for close analysis.

**Reviewer comment 4:**

*The paper draws eclectically from multiple theoretical traditions (postcolonial theory, trauma studies, memory studies, narrative ethics) without clearly articulating how these frameworks interact or which takes precedence when they might conflict. Add a brief theoretical framework subsection clarifying how you synthesize these different approaches.*

**Response:**

 We are grateful for this clarification request. In response, we have added a clear explanation of the theoretical framework in the revised manuscript. The paper now explicitly states that postcolonial theory functions as the primary interpretive lens, while trauma studies, memory studies, and narrative ethics are employed as complementary frameworks to illuminate psychological, ethical, and representational dimensions of postcolonial experience. These approaches are presented as intersecting rather than competing, allowing for a coherent synthesis across the analysis.

**Reviewer comment 5:**

*The conclusion could strengthen its case for why this comparison matters now. How does this analysis contribute to current debates in postcolonial studies? What does it offer readers?*

**Response:**

 We appreciate this suggestion and have revised the conclusion accordingly. The revised conclusion now explicitly situates the comparative analysis within contemporary postcolonial debates concerning migration, linguistic identity, and the reassessment of colonial legacies.

 To underscore the study’s contemporary relevance, we have added a brief discussion of recent educational reforms in Mali, where French colonial historical content has been suspended in secondary education. This example demonstrates that debates over colonial languages and literary canons remain globally relevant beyond Anglophone contexts, reinforcing the article’s broader argument about language, power, and postcolonial ethics.

 Once again, we thank the reviewer for their thoughtful engagement and constructive feedback. We believe that the revisions made in Version 2 address all major concerns and significantly strengthen the manuscript’s clarity, scholarly contribution, and contemporary relevance.
